# Deciphering the landscape of phosphorylated HLA-II ligands

**DOI:** 10.1016/j.isci.2022.104215

**Published:** 2022-04-06

**Authors:** Marthe Solleder, Julien Racle, Philippe Guillaume, George Coukos, Michal Bassani-Sternberg, David Gfeller

**Affiliations:** 1 Department of Oncology, Ludwig Institute for Cancer Research, University of Lausanne, 1011 Lausanne, Switzerland; 2 Swiss Institute of Bioinformatics (SIB), 1015 Lausanne, Switzerland; 3 Department of Oncology, University Hospital of Lausanne (CHUV), 1011 Lausanne, Switzerland

**Keywords:** Immunology, Cell biology, Omics

## Abstract

CD4^+^ T cell activation in infectious diseases and cancer is governed by the recognition of peptides presented on class II human leukocyte antigen (HLA-II) molecules. Therefore, HLA-II ligands represent promising targets for vaccine design and personalized cancer immunotherapy. Much work has been done to identify and predict unmodified peptides presented on HLA-II molecules. However, little is known about the presentation of phosphorylated HLA-II ligands. Here, we analyzed Mass Spectrometry HLA-II peptidomics data and identified 1,943 unique phosphorylated HLA-II ligands. This enabled us to precisely define phosphorylated binding motifs for more than 30 common HLA-II alleles and to explore various molecular properties of phosphorylated peptides. Our data were further used to develop the first predictor of phosphorylated peptide presentation on HLA-II molecules.

## Highlights

• 1,943 unique phosphorylated HLA-II ligands from MS HLA-II peptidomics data
• Binding motifs of phosphorylated HLA-II ligands identified for more than 30 alleles
• Predictor trained on phosphorylated peptides achieves higher accuracy

## Graphical abstract

**Figure:**
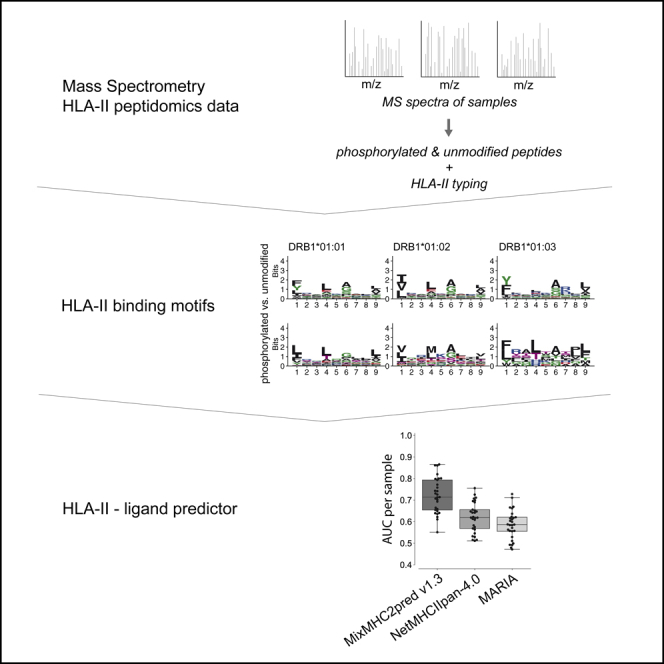

## Introduction

**Figure 1:**
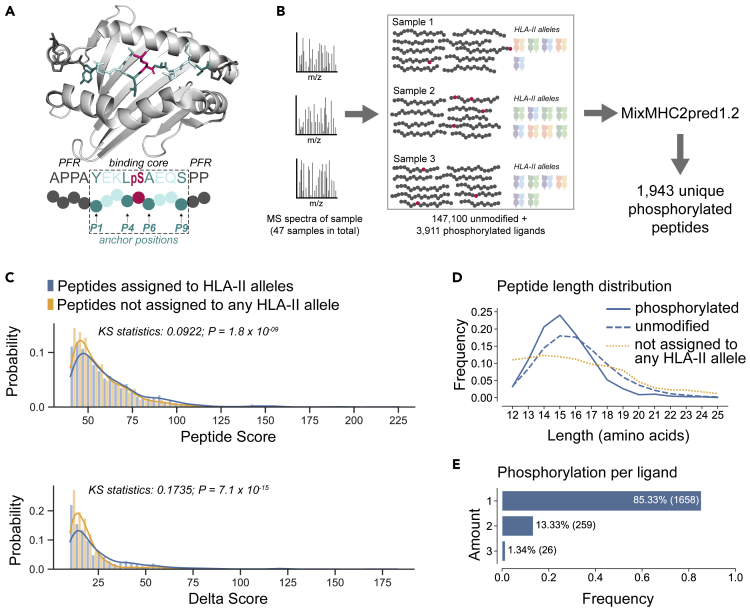
MS-based HLA-II peptidomics identifies multiple phosphorylated HLA-II ligands (A) Representative crystal structure of HLA-DRB1∗01:01 molecule in complex with a phosphorylated peptide (PDB identification code 3L6F (Li et al., 2010)). The binding core of the peptide is shown in turquoise, the peptide flanking regions (PFR) in dark gray, the phosphorylated residue in pink, and the HLA-DR in light gray. Anchor positions P1, P4, P6, and P9 are underlined in the peptide sequence and point toward the HLA-II binding site. (B) HLA-II peptidomics MS spectra were analyzed for each sample separately to identify HLA-II ligands, including phosphorylated peptides. Phosphorylated peptides were processed for each sample by applying the HLA-II ligand predictor MixMHC2pred. (C) Distribution of Andromeda search engine peptide spectrum match scores (*Peptide Score*) (top) and score differences to the second-best peptide spectrum match (*Delta Score*) (bottom) of phosphorylated peptides assigned to HLA-II alleles (blue) and phosphorylated peptides not assigned to any allele (orange). p-values were calculated using the Kolmogorov–Smirnov test. See also Figure S1. (D) Comparison of length distribution of phosphorylated and unmodified HLA-II ligands as well as predicted phosphorylated peptides not assigned to HLA-II alleles. (E) Amount of detected phosphorylated residues per phosphorylated HLA-II ligand in the HLA-II phosphopeptidome.

CD4^+^ T cells play a central role in adaptive immune responses against infections and cancer through the recognition of peptides coming from pathogens or specifically found in cancer cells. The latter include peptides originating from cancer specific genetic or proteomic alterations, often referred to as neo-antigens. Antigen presentation to CD4^+^ T cells is mediated by class II human leukocyte antigen (HLA-II) molecules, which are expressed on the surface of professional antigen-presenting cells (APCs) such as dendritic cells or B lymphocytes. HLA-II molecules form heterodimers and are encoded by three pairs of genes (HLA-DRA/B, HLA-DPA/B, and HLA-DQA/B). Except for HLA-DRA, these genes are highly polymorphic and thousands of alleles have been discovered in humans. HLA-II molecules bind mostly peptides of 12–20 amino acids with a 9-mer peptide binding core and flanking regions extending on both sides (see Figure 1A) (Chicz et al., 1992; Neefjes et al., 2011). For most alleles, the binding specificity is driven by the primary anchor residues at P1 and P9 and the secondary anchor residues at P4 and P6 of the peptide core, although some variability has been observed in anchor residues across different HLA-II alleles (Abelin et al., 2019; Racle et al., 2019). HLA-II ligands can originate from both exogenous and intracellular proteins processed by endocytic pathways (Roche and Furuta, 2015) and include both unmodified peptides as well as peptides with posttranslational modifications (PTMs) (Lim et al., 2021; Malaker et al., 2017). Recently, HLA-II ligands have been shown to play an important role in the response to personalized cancer vaccines (Alspach et al., 2019; Graciotti et al., 2020; Kranz et al., 2016; Kreiter et al., 2015; Sahin et al., 2017). HLA-II ligands can be identified either by Mass Spectrometry (MS) or using *in silico* predictions followed by experimental validation, although such experiments are technically challenging (Caron et al., 2017). Several predictors of HLA-II ligands have been developed (e.g., NetMHCIIpan-4.0 (Reynisson et al., 2020), MixMHC2pred (Racle et al., 2019), or MARIA (Chen et al., 2019)) and can contribute to reduce cost and efforts to identify novel HLA-II ligands, including class II neo-antigens. However, none of the existing predictors specifically integrate PTMs.

PTMs of proteins are essential regulators in many biological processes (Graves and Krebs, 1999; Hunter, 2009; Wang et al., 2014). PTMs like phosphorylation were shown to be deregulated in cancer cells, causing aberrant cellular behavior (Krueger and Srivastava, 2006; López-Otín and Hunter, 2010; Martín-Bernabé et al., 2017). Therefore, phosphorylated peptides presented on HLA molecules provide potential targets for the development of immunotherapeutic strategies (Engelhard et al., 2020; Lin et al., 2019; Meyer et al., 2009; Petersen et al., 2009a). Although many studies analyzed phosphorylated peptides presented on HLA-I molecules (Alpízar et al., 2017; Andersen et al., 1999; Cobbold et al., 2013; Mohammed et al., 2008; Petersen et al., 2009b; Refsgaard et al., 2021; Solleder et al., 2020; Zarling et al., 2006), phosphorylated HLA-II ligands have received much less attention. The first naturally presented phosphorylated HLA-II ligands were identified from an EBV-transformed B lymphoblastoid and a tumor cell line (Meyer et al., 2009). Shortly after, the first CD4^+^ T cell recognition of a phosphorylated HLA-II ligand was shown using the melanoma antigen Melan-A/MART-1 (Depontieu et al., 2009). Structural analysis of a phosphorylated peptide bound to HLA-DRB1 showed that the phosphorylated residue can in this case directly interact with the T cell receptor (Li et al., 2010). Although these studies provide evidences for HLA-II presentation of phosphorylated peptides and show potential applications as targets for immunotherapies, further characteristics such as binding motifs of phosphorylated HLA-II ligands on a large allelic coverage remain unknown and no HLA-II ligand predictor comprises phosphorylated peptides in its training set.

In this work, we reprocessed 47 high quality MS HLA-II peptidomics samples including phosphorylation in the spectral searches and identified 1,943 novel phosphorylated HLA-II ligands. Based on this data, we defined phosphorylated binding motifs of HLA-II alleles and identified specific molecular properties of phosphorylated HLA-II ligands. Furthermore, we developed the first HLA-II ligand prediction method specifically trained on phosphorylated peptides.

## Results

### MS-based HLA-II peptidomics identifies multiple phosphorylated HLA-II ligands

To identify a broad spectrum of phosphorylated HLA-II ligands across a wide range of HLA-II alleles, we reanalyzed raw MS HLA-II peptidomics data of 24 monoallelic samples (Abelin et al., 2019) and 23 polyallelic (Racle et al., 2019). 12 of the polyallelic samples were generated with HLA-DR and pan-HLA-II antibodies, one only with pan-HLA-II antibodies, and the other 10 only with pan-HLA-II antibodies (Table S1). We used MaxQuant, allowing for phosphorylation on serine, threonine, and tyrosine as variable modifications (see STAR Methods). To ensure broad coverage, we chose a loose false discovery rate (FDR) of 5% with restricted peptide identification scores (see STAR Methods). Across all samples, a total of 4,868 phosphorylated peptides (representing 3,717 unique peptides) were detected (Table S2). To determine HLA-II allelic restriction and remove expected contaminants or wrongly identified peptides, we performed predictions for each allele of each sample where the peptide was found using the HLA-II ligand predictor MixMHC2pred (Racle et al., 2019). For these predictions, phosphorylated residues were treated as glutamic acid in MixMHC2pred (see Figure 1B and STAR Methods). This resulted in 2,465 HLA-II-phosphorylated peptide interactions (representing 1,943 unique peptides, see Table S3). The other cases may consist of co-eluted contaminants or wrongly identified peptides, as expected in HLA-II peptidomics data (Racle et al., 2019). To support this hypothesis, we compared both the distribution of the scores for peptide spectrum matches from the Andromeda search engine (*Peptide Score*, higher values for higher confidence in peptide identification) and the distribution of the score differences to the second best peptide spectrum match (*Delta Score*, higher values for unambiguous distinction from other peptides) for peptides assigned to HLA-II alleles (blue in Figure 1C) and peptides that did not pass the MixMHC2pred filtering (orange in Figure 1C) (see also Figure S1). As expected, phosphorylated peptides that could not be assigned to any HLA-II allele showed lower *Peptide Scores* (KS statistics: 0.0922; p = 1.8 × 10^−9^) and lower *Delta Scores* (KS statistics: 0.1735; p = 7.1 × 10^−15^) than those that could be assigned to HLA-II alleles. These peptides were therefore excluded from downstream analyses. This represents roughly half (∼48%) of the peptides identified in our different samples. The same filtering applied to random peptides selected from a pool of all known phosphosites of the human proteome (Ullah et al., 2016), resulting in the exclusion of ∼70% of the peptides (see STAR Methods). This demonstrates that our set of phosphorylated peptides is enriched in peptides matching HLA-II motifs. The set of 1,943 unique phosphorylated HLA-II ligands showed a length distribution similar to the one of unmodified HLA-II ligands with a peak around 15-mers; however, the phosphorylated peptides that were not assigned to any allele and as a result excluded from downstream analyses had a length distribution skewed toward shorter peptides (Figure 1D). The majority of phosphorylated HLA-II ligands contained one phosphorylated residue, whereas ∼13.3% of the peptides were double phosphorylated and ∼1.3% were triple phosphorylated (Figure 1E).

### Phosphorylated peptides bind to HLA-II molecules with specific motifs

**Figure 2:**
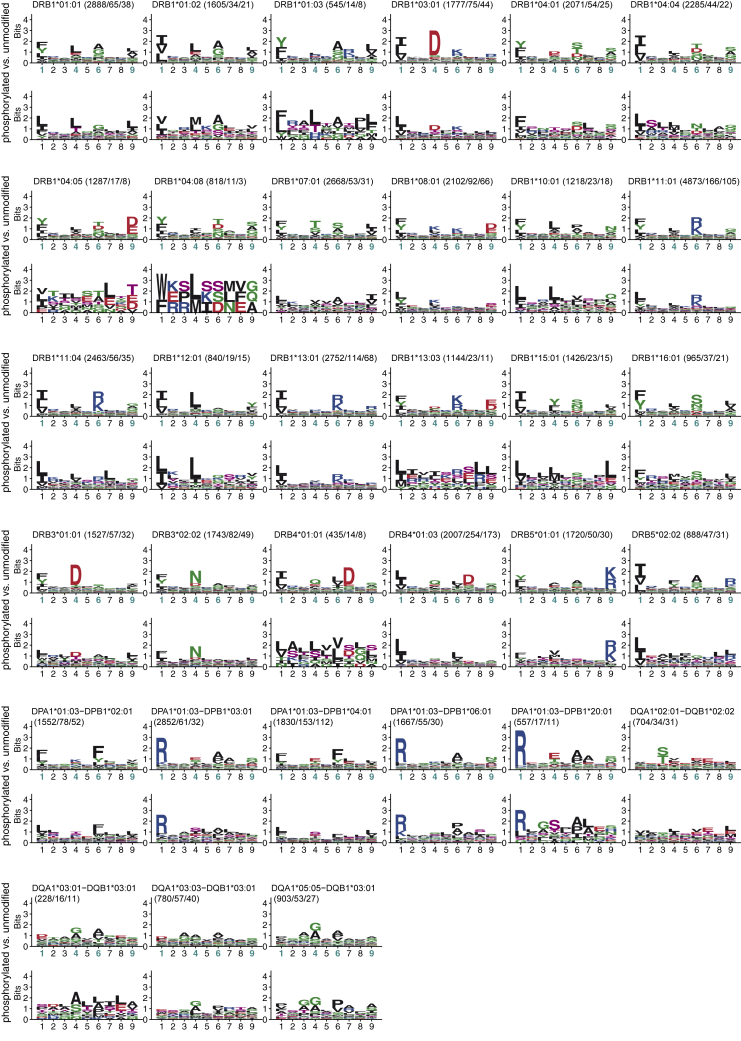
Phosphorylated peptides bind to HLA-II molecules with specific motifs Motifs of alleles with phosphorylated peptides. For each allele, the HLA-II motif based on unmodified ligands is shown on top, and the motif of phosphorylated HLA-II ligands determined in this work is shown below. Numbers correspond to the number of peptides (unmodified peptides/all phosphorylated peptides/only phosphorylated peptides with the phosphorylated residue in the core). Phosphorylated residues are shown in pink. Canonical anchor residues (P1, P4, P6, and P9) are highlighted in turquoise on the x-axis of binding motifs of unmodified ligands. See also Figure S2 and Table S3.

The 1,943 phosphorylated peptides could be assigned to 33 different alleles (Table S3). We then used the predicted binding cores to build binding motifs of phosphorylated HLA-II ligands for each of these alleles. Figure 2 shows that the binding motifs showed conserved specificity at anchor residues P1, P4, P6, and P9 for most alleles. To quantify this similarity, we compared the distances between pairs of unmodified and phosphorylated motifs of the same HLA-II alleles with one of the randomly paired alleles (see STAR Methods). Despite the known redundancy of HLA-II motifs, our results confirm that motifs of phosphorylated HLA-II ligands are significantly more similar to the one of unmodified ligands of the same allele, compared to the ones of other alleles (Figure S2).

Overall, we observed a slight enrichment of phosphorylated peptides in HLA-DQ ligands. Within HLA-DR alleles, HLA-DRB4 alleles displayed the highest frequency of phosphorylated ligands (Figure S3). HLA-DRB4 alleles have a preference for aspartic acid at P7, which suggests that the binding pocket favors negatively charged residues and is therefore suitable for phosphorylated residues at this position (Figure 2).

### Phosphorylated residues show positional specificity in HLA-II ligands

**Figure 3:**
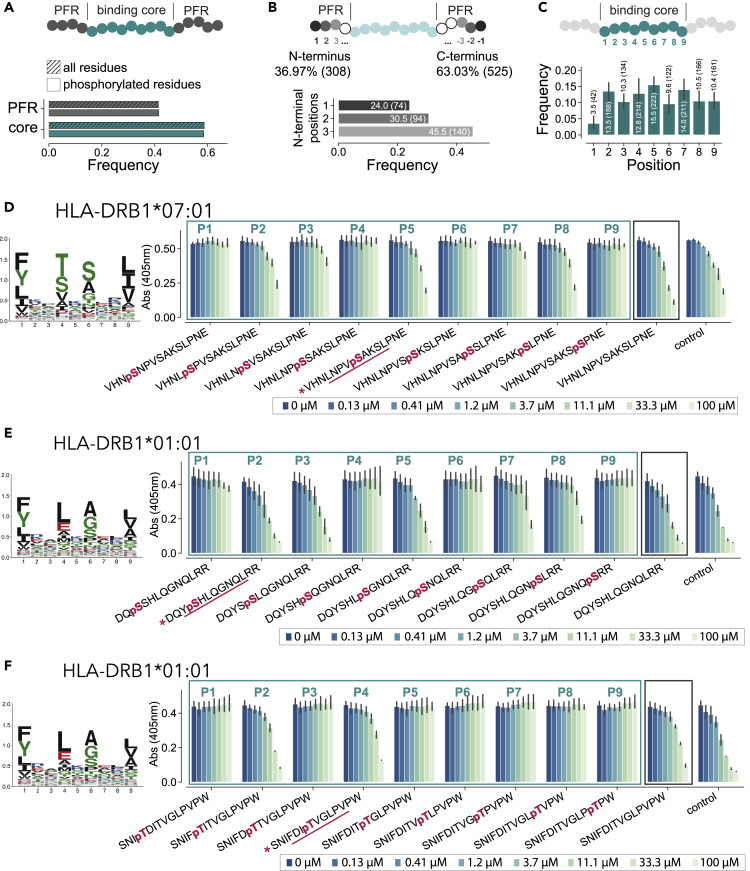
Phosphorylated residues show some positional specificity in HLA-II ligands (A) Distribution of phosphorylated residues and total residues in the binding core vs PFRs of phosphorylated HLA-II ligands. (B) Amount of phosphorylated residues found in the first three and last three residues in PFRs (top) and distribution of phosphorylated residues within the first three positions of the N-terminus (bottom) of phosphorylated HLA-II ligands. (C) Positional distribution of phosphorylated residues in the binding core of phosphorylated HLA-II ligands. Error bars represent the residue frequencies of individual alleles. (D–F) Competitor binding assays for peptides with a phosphorylated residue at each of the different core positions (turquoise box) and without phosphorylated residue (black box). The peptide initially found by MS is marked by a pink asterisk and the core predicted by MixMHC2pred is underlined. Error bars represent the two repetitions of the binding assays. HLA-II motifs of unmodified ligands of the respective allele are shown on the left.

To investigate whether there is any preference for phosphorylated residues in the peptide binding core and the peptide flanking regions (PFRs), we compared the fraction of phosphorylated residues with the total fraction of residues in these two regions of the peptide. We could see that the distribution of phosphorylated residues between the binding core and the PFRs is similar to the one of other amino acids (Figure 3A). We then analyzed phosphorylated residues in PFRs and compared their frequency in the first and last three amino acids of the PFRs at the N-terminus and C-terminus of the phosphorylated peptides. Phosphorylation sites occurred more frequently at the C-terminus of phosphorylated HLA-II ligands than at the N-terminus (with 63 and 37% of terminal phosphorylation found at C-terminus and at the N-terminus, respectively). We hypothesized that this could be because of the presence of clearer cleavage motifs at the N-terminus, and especially the preference for proline at the second position (Barra et al., 2018; Ciudad et al., 2017; Racle et al., 2019). This hypothesis is consistent with the distribution of phosphorylated residues within the N-terminal region where phosphorylation is mostly found at the third position (45.5%) and less at the other two (24 and 30.5%, respectively) (Figure 3B).

We then looked at the distribution of phosphorylated residues within the 9-mer binding core. We could clearly see less phosphorylated residues at the anchor position P1, which is consistent with the specificity for hydrophobic or positively charged amino acids observed in unmodified HLA-II ligands of most alleles (Figure 3C). The highest frequency of phosphorylated residues is seen at the non-anchor position P5, which shows low specificity in HLA-II binding motifs. Other positions such as at secondary anchor positions (especially P4 and P6) show more variability in the unmodified HLA-II binding motifs, which is also reflected by the presence of phosphorylated residues observed at these positions.

To further investigate the preference for phosphorylated residues at specific positions in the core, we performed competitor binding assays for two different HLA-DR alleles testing different versions of the same peptide containing the phosphorylated residues at all possible positions within the core (see STAR Methods). The two peptides were selected among the set of phosphorylated HLA-II ligands identified by MS with the phosphorylated residue at the non-anchor positions P2 and P5, respectively (Figures 3D and 3E). The results of the binding assays showed that for both alleles, the peptide that was found in our MS data displayed good binding (see Figure 3D for HLA-DRB1∗07:01 with pS at P5 and Figure 3E for HLA-DRB1∗01:01 with pS at P2). The unmodified version of the peptide was binding equally well. The presence of the phosphorylated residue at other positions showed inferior binding, especially at positions P1, P4, P6, and P9. These positions could clearly be identified as anchor positions of the alleles (see binding motifs Figures 3D and 3E left panels). We then selected another peptide for HLA-DRB1∗01:01 that was found in our MS data with a phosphorylated residue predicted at the secondary anchor position P4. The low binding with the phosphorylated residue at P1 and P9 could be confirmed. However, for other core positions, the results did not reflect the anchor residues and binding of this peptide was detected with a phosphorylated residue at P2 and P4 but not at P5 for instance (Figure 3F). Overall, these observations suggest that the preference for the position of the phosphorylated residue in the middle of the core may be different for different peptides, which could explain the relatively broad distribution in Figure 3C and the lack of exclusion of P4 and P6 secondary anchor positions.

### Kinase motifs in HLA-II ligands

**Figure 4:**
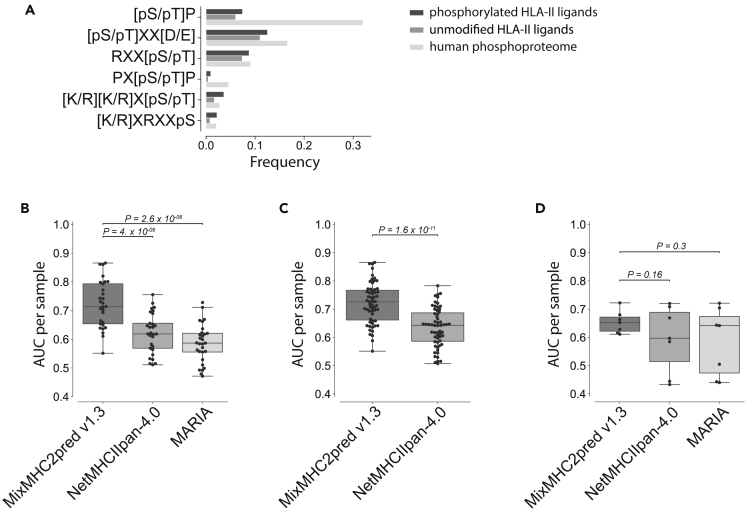
The HLA-II phosphopeptidome improves prediction of phosphorylated HLA-II ligands (A) Frequency of kinase motifs that show significant enrichment between phosphorylated and unmodified HLA-II ligands (p ≤ 0.05) in phosphorylated HLA-II peptidome (1^st^ bar), in unmodified HLA-II ligands (2^nd^ bar) and in the human phosphoproteome (3^rd^ bar). Kinase motifs are sorted according to the frequency in the human phosphoproteome. (B) AUC values for the leave-one-sample-out cross-validation for HLA-DR samples from (Racle et al., 2019) and (Abelin et al., 2019) and with available HLA-DR in MARIA (29 in total) for MixMCH2pred v1.3, NetMHCIIpred-4.0, and MARIA. (C) AUC values for the leave-one-sample-out cross-validation for all HLA-II samples from (Racle et al., 2019) and (Abelin et al., 2019) (59 in total) for MixMHC2pred v1.3 and NetMHCIIpan-4.0. (D) AUC values from the external validation for samples from (Khodadoust et al., 2017) for MixMHC2pred v1.3, NetMHCIIpan-4.0, and MARIA. p-values between the different predictors in (B–D) were calculated using the paired two-sided Wilcoxon signed rank-test. See also Figure S4.

To investigate the presence of kinase motifs in the HLA-II phosphopeptidome, we searched for known kinase motifs from the PhosphoMotif Finder of the Human Protein Reference Database (Amanchy et al., 2007) both in phosphorylated and unmodified HLA-II ligands as well as the human phosphoproteome (Sharma et al., 2014) (see STAR Methods). Motifs that show a significant enrichment between phosphorylated and unmodified HLA-II ligands (p ≤ 0.05) are shown in Figure 4A. These include frequent kinase motifs like [pS/pT]P, RXX[pS/pT], or [pS/pT]XX[D/E]. However, not all common kinase motifs were as enriched in our data as expected from the human phosphoproteome. For instance, the frequent kinase motif [pS/pT]P, which corresponds to proline-dependent serine/threonine kinases such as MAPK1, was only detected for 7.44% of phosphorylated serine and threonine in our phosphorylated HLA-II ligands. This number is higher than the frequency of proline after serine or threonine in the human proteome (6.78%) or in unmodified HLA-II ligands (6.03%) but still much lower than the observed frequency of proline after phosphorylated serine and threonine in the human phosphoproteome (32.02%). To identify the reasons for the limited enrichment of this kinase motif in the HLA-II phosphopeptidome, we investigated whether this may reflect a source protein bias in the HLA-II peptidome (i.e., peptides coming from proteins with such phosphorylation sites would be underrepresented in HLA-II ligands, irrespective of the phosphorylation status). To this end, we computed the overlap between the source genes of all HLA-II ligands and the source genes of proteins containing phosphosites with the [pS/pT]P motifs in the human phosphoproteome. This overlap was slightly higher than the expected one (odds ratio: 1.152), suggesting that there is no depletion of proteins containing the [pS/pT]P motifs in the HLA-II peptidome (see Figure S4A). This supports the idea that such phosphosites are mainly present in their unphosphorylated form in our set of HLA-II ligands.

### The HLA-II phosphopeptidome improves prediction of phosphorylated HLA-II ligands

We used the HLA-II phosphopeptidome to expand our HLA-II ligand prediction method (MixMHC2pred (Racle et al., 2019)) to phosphorylated peptides. To this end, MixMHC2pred v1.3 was retrained combining both unmodified and phosphorylated peptides (see STAR Methods). To benchmark its performance, we first performed a leave-one-sample-out cross-validation for each sample in our dataset. In each round of the cross-validation one sample was used as test set and the training of MixMHC2pred v1.3 was done with all phosphorylated peptides that could be assigned to HLA-II alleles, excluding those that were present in the sample used as test set (see STAR Methods). For each sample, phosphorylated peptides used as negatives were randomly selected from the human phosphoproteome, in 5-fold excess compared to the positives (see STAR Methods). Importantly, the sample used as a test set was not filtered with any predictor to prevent potential biases in the comparison between the different predictors. The area under the receiver operating characteristic curve (AUC) was used to assess the performance and to benchmark the new version of MixMHC2pred (v1.3) with the existing tools NetMHCIIpan-4.0 (Reynisson et al., 2020) and MARIA (Chen et al., 2019). In Figure 4B, we restricted to samples measured with HLA-DR specific antibodies, because MARIA predictions are limited to HLA-DR alleles. Both NetMHCIIpan-4.0 and MARIA are not specifically trained for modified residues, thus predictions of phosphorylated peptides with these tools were performed by substituting phosphorylated residues with ‘X’ for NetMHCIIpan-4.0 and by using the unmodified counterpart of the phosphorylated residue for MARIA (‘X’ are not supported in MARIA). Figure 4B shows improved predictions with MixMHC2pred v1.3. In Figure 4C, we considered data from all samples used in this work and restricted the comparison to NetMHCIIpan-4.0, because MARIA can only be applied on HLA-DR alleles. Here again, MixMHC2pred v1.3 displayed improved accuracy. Results did not change if we used Matthew’s correlation coefficient or F1 score instead of AUC (Figures S4B and S4C).

As a second external validation, we reprocessed MS data from (Khodadoust et al., 2017), searching for phosphorylated HLA-II ligands (see STAR Methods). We then investigated how accurately these phosphorylated HLA-II ligands would be predicted by MixMHC2pred v1.3, NetMHCIIpan-4.0, and MARIA. This external benchmark confirmed the improved predictions with MixMHC2pred v1.3, although comparison of AUC, Matthew’s correlation coefficient or F1 scores did not reach statistical significance on these seven samples (Figures 4D and S4D).

## Discussion

A better understanding of the repertoire and the properties of HLA-II ligands is promising for the development of personalized cancer immunotherapies such as cancer vaccines (Alspach et al., 2019; Kranz et al., 2016; Kreiter et al., 2015; Sahin et al., 2017). As cancer can cause aberrant PTMs, large datasets of modified HLA-II ligands informing us about the rules for the presentation of such ligands and enabling us to predict them are useful to expand the list of potential targets for cancer immunotherapy. It is also likely that several phosphorylated peptides from pathogens are displayed on HLA-II molecules, although little data is available about them, partly because of the previous lack of predictors for such ligands.

In this work, we performed an in-depth analysis of the HLA-II phosphopeptidome. We could identify binding motifs of phosphorylated HLA-II ligands for more than 30 alleles. These binding motifs showed high similarity with those of unmodified HLA-II ligands at anchor positions, in particular the main anchors at P1 and P9.

Our analysis of the position of phosphorylated residues in HLA-II ligands revealed a preference for phosphorylation in the middle of the core (P5) and low frequency of phosphorylated residues at the anchor position P1 (Figure 3C). These results could be confirmed with binding assays and are consistent with the low frequency of phosphorylated residues at anchor positions in HLA-I ligands (Solleder et al., 2020). The presence of phosphorylated residues at secondary anchor positions (mainly P4 and P6) was less expected. However, our binding assays confirmed that specific alleles can accommodate phosphorylated residues at such secondary anchor positions (Figure 3F). The lower frequency of phosphorylated residues at the N-terminus compared to the C-terminus of the HLA-II ligands as well as the depletion of phosphorylation at the first and second positions of the N-terminus (Figure 3B) suggest that the presence of phosphorylated residues at these positions may not be favorable for protein cleavage or transport into the ER, although additional work will be needed to validate this hypothesis.

Our analysis of kinase motifs detected overrepresentation of only a few known kinase motifs. Although, the very frequent [pS/pT]P motif was seen at a higher frequency in the phosphorylated HLA-II peptidome compared to the unmodified HLA-II peptidome, its frequency was less than what would be expected from the human phosphoproteome. We speculate that many of these potential phosphosites are simply not phosphorylated in the pool of ligands available for loading onto HLA-II molecules or that the phosphate groups are removed by phosphatases before or after binding to the HLA-II molecules. The limited enrichment in motifs for intracellular kinases also supports the idea that phosphorylated residues observed among HLA-II ligands come from a more diverse repertoire of kinases compared to the one observed in the HLA-I phosphopeptidome (Solleder et al., 2020). This hypothesis is consistent with the differences between class I and class II antigen presentation pathways and the fact that many HLA-II ligands come from endocytosis of proteins in the extracellular matrix, which may undergo phosphorylation by different sets of kinases compared to intracellular proteins displayed on HLA-I molecules.

To facilitate further studies of phosphorylated HLA-II ligands, we used our data to build a predictor for phosphorylated HLA-II ligands by including the HLA-II phosphopeptidome in the training data of our HLA-II ligand prediction method MixMHC2pred (v1.3). Our results show that this new predictor has higher accuracy compared to other tools that did not include phosphorylated HLA-II ligands in their training (Figures 4B–4D and S4B–S4D). The motifs of phosphorylated HLA-II ligands suggest that the binding of phosphorylated peptides is shaped by the binding motif of the HLA-II allele and some positional specificity for the phosphorylated residues (e.g., exclusion of P1), and that this information is accurately captured by MixMHC2pred v1.3.

Altogether, our work represents the first in-depth analysis of the repertoire of phosphorylated HLA-II ligands. We anticipate that this resource and the associated computational tools to predict phosphorylated HLA-II ligands in different contexts will facilitate the discovery of potential new targets for CD4^+^ T cell recognition in infectious diseases and cancer immunotherapy.

### Limitations of the study

One important limitation in our work is the use of HLA-II ligand predictors to filter potential contaminants and assign allelic restrictions to the phosphorylated peptides seen in MS HLA-II peptidomics data. Although HLA-II ligand predictors are often used to filter HLA-II peptidomics data (Marcu et al., 2021), we cannot exclude that some phosphorylated peptides would bind to HLA-II molecules with a different binding mode that is not found in unmodified ligands. An alternative would be to use motif deconvolution tools like MoDec (Racle et al., 2019). Unfortunately, we could not obtain reliable and robust results with MoDec on our data. This is mainly because MoDec is sensitive to the background frequency of amino acids. These frequencies are very difficult to estimate for phosphorylated residues and small changes in these frequencies had a big impact on the phosphorylated HLA-II motifs. For this reason, we had to rely on HLA-II ligand predictions.

## STAR★Methods

### Key resources table

**Table:** 

REAGENT or RESOURCE	SOURCE	IDENTIFIER
**Antibodies**		

Anti-Flag Alkaline Phosphatase	Sigma-Aldrich	Cat# A9469; RRID:AB_439699

**Chemicals, peptides, and recombinant proteins**		

Influenza HA_307-319_	Home made	N/A
NY-ESO-1_87-99_	Home made	N/A
Avidin	Sigma-Aldrich	https://www.sigmaaldrich.com/CH/fr/product/sigma/a9275
pNPP	Sigma-AldrichMerck	https://www.sigmaaldrich.com/CH/fr/product/sigma/n2770
HLA-DRB1∗01:01/HLA-DRB1∗07:01	Home made	N/A

**Software and algorithms**		

BioPython	Python library	https://biopython.org/
Pandas	Python library	https://pandas.pydata.org/
Seaborn	Python library	https://seaborn.pydata.org/
SciPy	Python library	https://scipy.org/
Scikit-learn	Python library	https://scikit-learn.org
Ggseqlogo	Wagih (2017)	https://github.com/GfellerLab/ggseqlogo
MixMHC2pred v1.2	Racle et al. (2019)	https://github.com/GfellerLab/MixMHC2pred
MixMHC2pred v1.3	This study	https://github.com/GfellerLab/MixMHC2pred/tree/MixMHC2pred1.3
NetMHCIIpan-4.0	Reynisson et al. (2020)	https://services.healthtech.dtu.dk/service.php?NetMHCIIpan-4.0
MARIA	Chen et al. (2019)	https://maria.stanford.edu/

**Other**		

Mass Spectrometry peak lists of 23 multiallelic samples	Racle et al. (2019)	https://www.ebi.ac.uk/pride/archive/projects/PXD012308
Mass Spectrometry peak lists of 24 monoallelic samples	Abelin et al. (2019)	ftp://massive.ucsd.edu/MSV000083991
Mass Spectrometry peak lists of external benchmarking dataset	Khodadoust et al. (2017)	https://www.ebi.ac.uk/pride/archive/projects/PXD004746

### Resource availability

#### Lead contact

Further information and requests for resources and reagents should be directed to and will be fulfilled by the lead contact, David Gfeller (david.gfeller@unil.ch).

#### Materials availability

This study did not generate new unique reagents.

### Method details

#### Curation of immunopeptidomics HLA-II MS datasets

The MaxQuant platform (Cox and Mann, 2008) version 1.5.5.1 was employed to search the MS peak lists of 23 multiallelic samples from (Racle et al., 2019), 24 monoallelic samples from (Abelin et al., 2019). 10 multiallelic samples were processed only with pan-HLA-II antibodies, 12 with both HLA-DR and pan-HLA-II antibodies, and 1 multiallelic sample was only processed with HLA-DR antibodies (Table S1). The search was performed against a fasta file containing the human proteome (UniProt: UP000005640, reviewed, with no isoforms, including 21,026 entries downloaded in March 2017) and a list of 247 frequently observed contaminants. Peptides with a length between 8 and 25 amino acids were allowed. The second peptide identification option in Andromeda was enabled and the enzyme specificity was set as unspecific. An FDR of 5% was required for peptides and no protein false-discovery rate was set. The initial allowed mass deviation of the precursor ion was set to 6 ppm and the maximum fragment mass deviation was set to 20 ppm. Methionine oxidation, N-terminal acetylation and phosphorylation on serine, threonine, and tyrosine were set as variable modifications. The resulting list of msms identifications were further filtered to include phosphorylated peptides with identification score ≥40, score difference to the second best peptide spectrum match (*Delta Score*) ≥ 10, and localization probability for phosphorylation of >0.75 as well as peptide lengths restricted to 12 to 25 amino acids (Table S2).

Unmodified HLA-II ligands from the monoallelic samples in (Abelin et al., 2019) were obtained using the same MS search pipeline as for the phosphorylated peptides. Unmodified HLA-II ligands from other samples used in the training of MixMHC2pred v1.3 were retrieved directly from (Racle et al., 2019).

Phosphorylated peptides were also searched in seven samples from (Khodadoust et al., 2017) (Table S2) using the approach described above, and these were only used as an external dataset to benchmark MixMHC2pred v1.3.

#### Peptide filtering, allele assignment, and core prediction using the HLA-II ligand predictor

To filter potential contaminants or wrongly identified peptides and to determine allelic restriction and peptide binding cores, the HLA-II ligand predictor MixMHC2pred (v1.2) (Racle et al., 2019) was applied to all phosphorylated peptides for all alleles available in each of the 47 sample (see Table S1 for HLA typing of each sample). Phosphorylated residues were substituted by glutamic acid. To filter potential contaminants or wrongly identified peptides, a %rank cutoff of 10% was applied to the data. Peptides not passing this threshold were not considered in any analysis. The same predictions were performed on random phosphorylated peptides selected from a pool of all known phosphosites of the human proteome (Ullah et al., 2016) with the same length distribution as the phosphorylated HLA-II ligands found by MS. Sequence logos including phosphorylated peptides were drawn with the extended version of ggseqlogo (https://github.com/GfellerLab/ggseqlogo) (Wagih, 2017) and phosphorylated residues are shown in purple (Figure 2). Phosphorylated HLA-II binding motifs shown in Figure 2 were built using only peptides containing phosphorylated residues within the binding core.

To compare binding motifs of phosphorylated and unmodified ligands, Euclidean distances were computed between position weight matrices of phosphorylated (PWM_phos_) and unmodified (PWM_unmod_) HLA-II ligands. For a meaningful comparison, only unmodified residues were considered in PWM_phos_ and the matrices were renormalized accordingly. For intra-allele distances, the distance was measured between PWM_phos_ and PWM_unmod_ of the same allele. For inter-allele distances, distances between PWM_phos_ and PWM_unmod_ of all 32 other alleles was computed (Figure S2).

#### Positional distribution of phosphorylated residues in HLA-II ligands

The frequency of phosphorylated residues inside of the binding core and in PFRs were compared to the total fraction of residues in these two regions of the peptides (Figure 3A). Only peptides with phosphorylated residues in the first three positions of the N-terminal region or in the last three positions of the C-terminal region were used to compute the distribution of phosphorylated residues in PFRs (Figure 3B). The distribution of phosphorylated residues per position in the core was computed position-wise for all peptides that contained at least one phosphorylation in the binding core (Figure 3C).

#### Competition binding assays

To test binding of different phosphorylated HLA-II ligands, competition assays were performed for HLA-DRB1∗01:01 and HLA-DRB1∗07:01 with two and one different peptides detected by MS in the samples, respectively. The competition assays were performed by mixing in v-bottom 96-well plate (Greiner Bio-One) in a citrate saline buffer (100 mM citrate, pH 6.0), with 0.2% β-octyl-glucopyranoside (Calbiochem), 1×complete protease inhibitors (Roche), and 1 μg of the biotinylated empty allele with an FLAG-tagged peptide at fixed concentration of 2 μM (Influenza HA_307-319_ for HLA-DRB1∗01:01 and NY-ESO-1_87-99_ for HLA-DRB1∗07:01). The peptide of interest was added to this mix into each well at a final concentration of 0, 0.13, 0.41, 1.3, 3.7, 11.1, 33.3, and 100 μM. For the control, untagged peptide (Influenza HA_307-319_ or NY-ESO-1_87-99_) were added at the respective concentrations to the mix of allele and FLAG-tagged peptide. After incubation at 37°C overnight, the binding of the tagged peptides to HLA-II molecule was measured by ELISA. The mix was transferred to a plate coated with avidin and the FLAG-peptide was detected with an anti-FLAG-alkaline phosphatase conjugate (Sigma), developed with pNPP SigmaFAST substrate and absorbance was read with a 405nm – filter (Figures 3D–3F).

#### Kinase motifs

To detect enrichment of kinase motifs in phosphorylated HLA-II ligands, occurrences of all motifs from the PhosphoMotif Finder of the Human Protein Reference Database (Amanchy et al., 2007) were searched in phosphorylated as well as unmodified HLA-II ligands (Figure 4A). To be able to search each motif on all peptides, including those that had the phosphorylated residue at the first or last positions, each phosphorylated and unmodified peptide was mapped to its source protein and N- and C-terminally extended. To compute frequencies in Figure 4A, occurrences of kinase motifs were normalized by the amount of phosphorylated residues of the corresponding motif in all phosphorylated peptides (e.g., amount of pS and pT in all phosphorylated peptides for motif [pS/pT]P). Similarly, frequencies of kinase motifs in unmodified peptides were determined by normalization with the amount of the unmodified counterpart of the phosphorylated residues of the corresponding motif in all unmodified peptides (e.g., amount of S and T in unmodified peptides for motif [S/T]P). For comparison, the same analysis was also performed on the human phosphoproteome (Sharma et al., 2014). The most common and non-redundant kinase motifs that showed a p-value ≤ 0.05 between phosphorylated and unmodified HLA-II peptides (computed with one-sided Fisher’s exact test) are shown in Figure 4A. To analyze whether the difference in kinase motifs between phosphorylated HLA-II and the human phosphoproteome (Sharma et al., 2014) is due to a gene bias of source proteins, a universal set of source genes of MS-detected sequences was defined. This universal gene set contained all source genes of phosphorylated and unmodified HLA-II sequences, source genes from a phosphoproteome (Sharma et al., 2014) and an MS-based human proteome (Wilhelm et al., 2014). Next, source genes of known phosphosites from the phosphoproteome containing the [pS/pT]P motif were identified and the overlap with unmodified HLA-II ligands was computed (see Figure S4A).

#### Predictor

Predictions of interactions between HLA-II alleles and phosphorylated peptides were based on the previously developed HLA-II ligand prediction method MixMHC2pred (Racle et al., 2019). Following our previous work on phosphorylated HLA-I ligands (Solleder et al., 2020), the MixMHC2pred training framework was extended to consider 23 amino acids. The phosphorylated peptides as well as unmodified peptides from the monoallelic samples (Abelin et al., 2019) were added to the training set used in (Racle et al., 2019). MixMHC2pred v1.3 was then retrained on this combined dataset of both phosphorylated and unmodified HLA-II ligands. For the leave-one-sample-out cross-validation (Figures 4B, 4C, S4B, and S4C), each sample from the dataset was iteratively used as test set and all phosphorylated peptides that were found in this sample were removed from the training data of MixMHC2pred v1.3. The remaining phosphorylated peptides were then added to the unmodified HLA-II ligands and MixMHC2pred v1.3 was retrained at each step of the cross-validation. Five times the amount of positive phosphorylated peptides were added to the testing data as negative peptides. Peptides used as negatives in the test set were of lengths 12 to 25 amino acids and contained a phosphosite from a pool of all known phosphosites of the human proteome (Ullah et al., 2016) (the phosphosite itself, the length of the peptide as well as the position of the phosphosite in the 12 to 25-mers were randomly chosen). The same way of adding negatives was applied to samples from the external dataset of phosphorylated peptides from (Khodadoust et al., 2017) used to provide an independent validation of the predictor.

Other existing HLA-II predictors (MARIA (Chen et al., 2019) and NetMHCIIpan-4.0 (Reynisson et al., 2020)) were used to benchmark the prediction results (Figures 4B–4D and S4B–S4D). MARIA was used with the unmodified version of the phosphorylated peptides (S, T, Y instead of pS, pT, pY) as well as gene names of the peptides’ source proteins and only applied to HLA-DR alleles. Phosphorylated residues in HLA-II ligands were substituted by ‘X’ for predictions with NetMHCIIpan-4.0. For the prediction of peptides coming from samples measured also with pan-HLA-II specific antibodies, alleles available in MixMHC2pred v1.3 were used for both MixMHC2pred v1.3 and NetMHCIIpan-4.0. For comparison of the predictions with each method, the area under the curve (AUC) of the receiver operating characteristic (ROC) was computed for each sample and each predictor (Figures 4B–4D). Additionally, the Matthew’s correlation coefficient, using a %rank threshold of 5% to classify binders and non-binders, and the F1 score were calculated for each sample and each predictor (Figures S4B–S4D).

### Quantification and statistical analysis

Statistical analyses of this study were performed using Python libraries SciPy and Scikit-learn and p-values ≤ 0.05 were considered significant. Statistical significances were analyzed with two-sample Kolmogorov-Smirnov tests (distribution of Peptide Score and Delta Score), Wilcoxon rank sum tests (similarity phosphorylated and unmodified HLA-II binding motifs), two-sample t-tests (amount phosphorylated peptides per allele), and one-sided Fisher’s exact tests (identifying enriched kinase motifs). Classification metrices measuring accuracy of predictors were compared with Wilcoxon signed-rank tests. Boxplots were created with the Python library Seaborn, using default parameters for defining interquartile ranges and outliers.

## Data Availability

• This paper analyzes existing, publicly available data. These accession numbers for the datasets are listed in the Key resources table. • The command-line script to run the new version the HLA-II ligand prediction method (MixMHC2pred v1.3) is available at https://github.com/GfellerLab/MixMHC2pred/tree/MixMHC2pred1.3. • Any additional information required to reanalyze the data reported in this paper is available from the Lead contact upon request.

## Supplemental information

Document S1. Figures S1–S4

Table S1: List of all samples from (Racle et al., 2019), (Abelin et al., 2019), and (Khodadoust et al., 2017) (Sheet, 1, 2, and 3, respectively) used in this work, related to STAR Methods The first column gives information about the sample and the second column corresponds the HLA typing of each sample as well as the HLA alleles available for predictions with MixMHC2pred.

Table S2: MS/MS data for all phosphorylated peptides used in this study, related to STAR Methods Sheet 1: The list contains phosphorylated peptides of length 12 to 25, identified across all 23 multiallelic samples from (Racle et al., 2019) and all 24 monoallelic samples from (Abelin et al., 2019). Sheet 2: The list contains phosphorylated peptides of length 12 to 25, identified across 7 samples from (Khodadoust et al., 2017). For all searchers a Peptide Score ≥40, a Delta Score ≥10, and a localization probability for phosphorylation >0.75 was applied. Phosphorylated residues are given in lowercase letters.

Table S3: List of phosphorylated peptides that were assigned to HLA-II alleles, related to STAR Methods The second column indicates the allele(s) to which the phosphorylated peptide is predicted to bind. The third column shows the predicted core(s). Phosphorylated residues are given in lowercase letters.
