# Supplementary material for: Deciphering the landscape of phosphorylated HLA-II ligands
Source: iScience. 2022 Apr 6;25(5):104215. doi: 10.1016/j.isci.2022.104215 (PMC9051626; doi:10.1016/j.isci.2022.104215)
Supplement: Document S1. Figures S1–S4 [file mmc1.pdf]

iScience, Volume *25*

Supplemental information

Deciphering the landscape

# of phosphorylated HLA-II ligands

Marthe Solleder, Julien Racle, Philippe Guillaume, George Coukos, Michal Bassani-Sternberg, and David Gfeller

Supplemental Information

Supplementary Figures

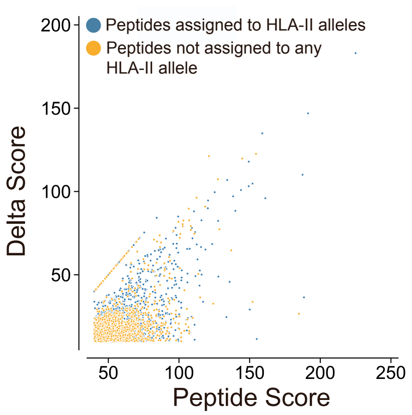

Figure S1: Plot of Andromeda search engine peptide spectrum match scores (*Peptide Score*) vs. score differences to the second-best peptide spectrum match (*Delta Score*) of the phosphorylated peptides, related to Figure 1. Peptides assigned to HLA-II alleles are shown in blue, the others are shown in orange.

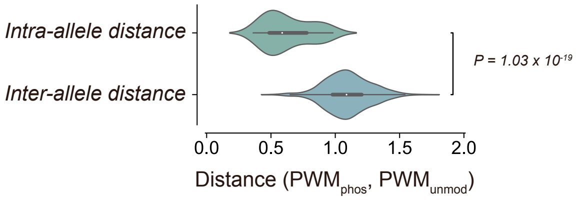

Figure S2: Similarity between phosphorylated and unmodified HLA-II binding motifs, related to Figure 2. Euclidean distances between PWMs of phosphorylated ligands and PWMs of unmodified ligands for the same allele (*intra-allele distance*, top panel) and for different alleles (*inter-allele distance*, bottom panel) are shown.

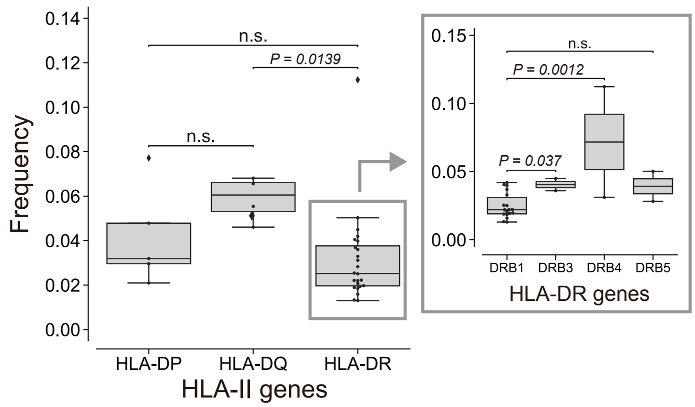

Figure S3: HLA-II alleles present similar fractions of phosphorylated ligands, related to Figure 2. Frequency of phosphorylated peptides in the HLA-II peptidome for the alleles of each gene (HLA-DRB1/3/4/5, -DP, or -DQ).

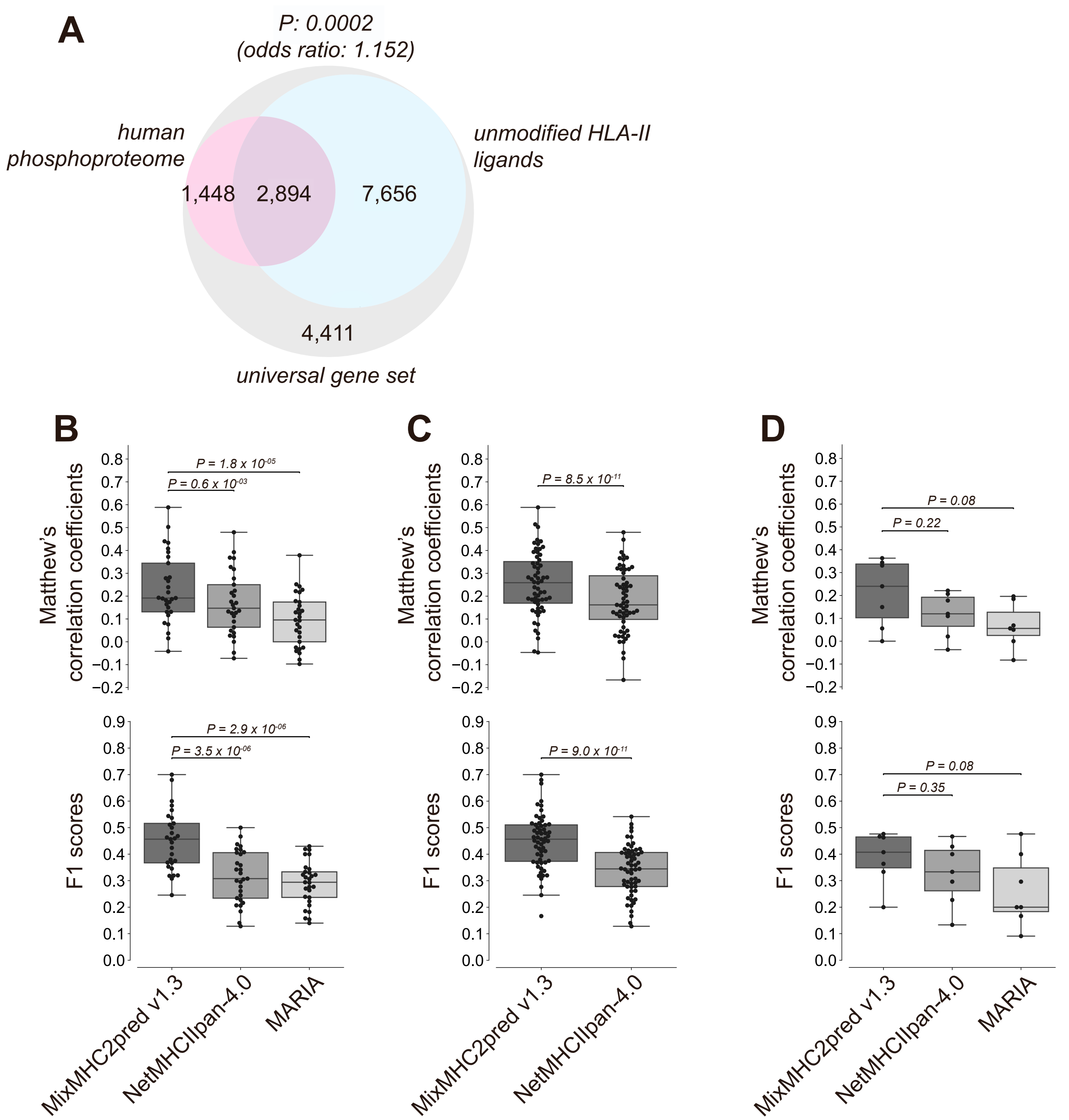

Figure S4: The HLA-II phosphopeptidome improves prediction of phosphorylated HLA-II ligands, related to Figure 4. (A) Overlap between source genes of proteins containing phosphosites with the [pS/pT]P motifs in the human phosphoproteome (pink) and source genes of all HLA-II ligands (light blue). **(B)** Matthew's correlation coefficients (upper panel) and F1 scores (lower panel) for the leave-one-sample-out cross-validation for HLA-DR samples from (Racle et al., 2019) and (Abelin et al., 2019) and with available HLA-DR in MARIA for MixMCH2pred v1.3, NetMHCIIpred-4.0, and MARIA. **(C)** Matthew's correlation coefficients (upper panel) and F1 scores (lower panel) for the leave-one-sample-out cross-validation for all HLA-II samples from

(Racle et al., 2019) and (Abelin et al., 2019) for MixMHC2pred v1.3 and NetMHCIIpan-4.0. **(D)** Matthew’s correlation coefficients (upper panel) and F1 scores (lower panel) from the external validation for samples from (Khodadoust et al., 2017) for MixMHC2pred v1.3, NetMHCIIpan-4.0, and MARIA.
